# Exploring the Impact of Irradiation on Glioblastoma Blood-Brain-Barrier Permeability: Insights from Dynamic-Contrast-Enhanced-MRI and Histological Analysis

**DOI:** 10.3390/biomedicines12051091

**Published:** 2024-05-14

**Authors:** Jérôme Conq, Nicolas Joudiou, Véronique Préat, Bernard Gallez

**Affiliations:** 1 Biomedical Magnetic Resonance Research Group, Louvain Drug Research Institute (LDRI), Université Catholique de Louvain (UCLouvain), 1200 Brussels, Belgium; jerome.conq@uclouvain.be; 2 Advanced Drug Delivery and Biomaterials Research Group, Louvain Drug Research Institute (LDRI), Université Catholique de Louvain (UCLouvain), 1200 Brussels, Belgium; veronique.preat@uclouvain.be; 3 Nuclear and Electron Spin Technologies (NEST) Platform, Louvain Drug Research Institute (LDRI), Université Catholique de Louvain (UCLouvain), 1200 Brussels, Belgium; nicolas.joudiou@uclouvain.be

**Keywords:** blood-brain barrier, glioblastoma, radiotherapy, DCE-MRI, diffusion-MRI, Evans blue

## Abstract

(1) Background: Glioblastoma (GB) presents a formidable challenge in neuro-oncology due to its aggressive nature, limited treatment options, and poor prognosis. The blood–brain barrier (BBB) complicates treatment by hindering drug delivery to the tumor site, particularly to the infiltrative cells in the margin of the tumor, which are mainly responsible for tumor recurrence. Innovative strategies are therefore needed to enhance drug delivery in the margins of the tumor. This study explores whether irradiation can enhance BBB permeability by assessing hemodynamic changes and the distribution of contrast agents in the core and the margins of GB tumors. (2) Methods: Mice grafted with U-87MG cells were exposed to increasing irradiation doses. The distribution of contrast agents and hemodynamic parameters was evaluated using both non-invasive magnetic resonance imaging (MRI) techniques with gadolinium–DOTA as a contrast agent and invasive histological analysis with Evans blue, a fluorescent vascular leakage marker. Diffusion–MRI was also used to assess cytotoxic effects. (3) Results: The histological study revealed a complex dose-dependent effect of irradiation on BBB integrity, with increased vascular leakage at 5 Gy but reduced leakage at higher doses (10 and 15 Gy). However, there was no significant increase in the diffusion of Gd-DOTA outside the tumor area by MRI. (4) Conclusions: The increase in BBB permeability could be an interesting approach to enhance drug delivery in glioblastoma margins for low irradiation doses. In this model, DCE-MRI analysis was of limited value in assessing the BBB opening in glioblastoma after irradiation.

## 1. Introduction

Glioblastoma (GB) is a highly aggressive and malignant brain cancer that remains a formidable challenge in the realm of neuro-oncology, with limited treatment options and a poor prognosis. The current therapeutic approach (standard of care) for GB involves a combination of surgical resection, radiation therapy (fractionated focal irradiation in daily fractions of 2 Gy given 5 days per week for 6 weeks, for a total of 60 Gy), and chemotherapy, commonly employing temozolomide (TMZ, 75 mg per square meter of body-surface area per day, 7 days per week from the first to the last day of radiotherapy) [1]. Despite these efforts, the prognosis for GB remains bleak, largely due to the tumor’s infiltrative nature and high recurrence rates, with a median survival of around 15 months post diagnosis [2,3]. This cancer is characterized by rapid growth, a high degree of cellular and genetic heterogeneity, resistance to chemotherapy, and highly invasive properties with infiltrative cells that spread into the surrounding brain tissues, making complete surgical removal challenging and contributing to high rates of recurrence [4].

**Figure 1:**
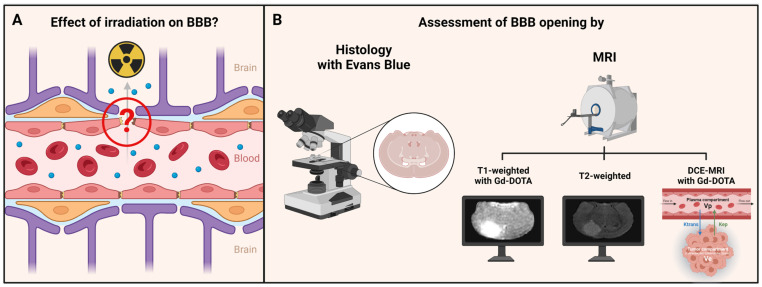
Assessment of the impact of irradiation on the BBB. (**A**) When not exposed to irradiation, a non-fenestrated BBB (as in tumor margins) has a very low permeability. The purpose here was to assess the effect of irradiation on the BBB. (**B**) This assessment was carried out by histological analysis with Evans blue and MRI techniques (T_1_-weighted, T_2_-weighted, and dynamic-contrast-enhanced MRI) with gadolinium–DOTA as contrast agent. Figure created with Biorender.com. License to B.G.

The intrinsic resistance of GB to conventional treatments is exacerbated by the blood–brain barrier (BBB), hindering effective drug delivery to the tumor site. The BBB is a highly selective, semipermeable barrier that regulates the passage of substances between the bloodstream and the brain to protect the brain from potentially harmful agents, including toxins, pathogens, and, of interest here, drugs while maintaining an optimal microenvironment for neural function [5]. In glioblastoma, although the tumoral BBB is leaky due to a paracellular pathway that is damaged, its permeability is highly heterogeneous and varies not only between GBs but also within the same tumor, leading to a perfusion and a consequent heterogeneous delivery of consequent heterogeneous delivery of drugs [6]. Indeed, the BBB is often compromised in the tumor core, allowing the passage of drugs, but in the margins of the tumor, the barrier is still intact or only slightly compromised, significantly limiting the passage of drugs, especially for reaching the infiltrating tumor cells that will be at the origins of the tumor recurrence [7,8,9,10]. Thus, there is an urgent need to develop methods for an opening of the BBB so that drugs can be delivered evenly to the tumor site. Recognizing this challenge, emerging research explores innovative approaches, such as irradiation-induced disruption of the BBB. Indeed, previous works have shown that irradiation can induce an opening in the healthy BBB or tumor vascular bed in other cancers to glioblastoma [11,12,13], or even increase drug delivery in glioblastoma [14,15], reflecting an opening of the BBB. This article explores if irradiation could open the tumoral BBB in GB, with a special focus on the margins of the tumor, providing a promising avenue for enhanced drug delivery, and therefore improved therapeutic efficacy (Figure 1). Indeed, as radiotherapy is included in the standard therapeutic protocol, this would give us a better understanding of its impact on the delivery/diffusion of the concomitant chemotherapy, and enable us to assess whether there is an irradiation dose for which diffusion would be the most ideal, to adapt the protocol of concomitant chemotherapy in the future.

For this purpose, we evaluated whether a radiotherapy protocol may change hemodynamic parameters in the core and the margins of the tumor in a GB model. Two methods have been used to evaluate the efficacy of the treatment on perfusion/permeability parameters (Figure 1): a non-invasive one, based on magnetic resonance imaging (MRI) analysis (T_1_-weighted and T_2_-weighted imaging as well as dynamic-contrast-enhanced (DCE)—MRI), and an invasive one, based on histological analysis with a fluorescent vascular leakage marker (Evans blue, EB).

For the MRI analysis, the tumor core was determined using T_2_-weighted contrast images. Moreover, T_1_-weighted images were acquired following the administration of a contrast agent to assess the diffusion of the contrast agent within both the bulk of the tumor and its margins. Gadolinium–DOTA was selected as a contrast agent due to its inability to cross the intact blood–brain barrier (BBB), providing an avenue to investigate changes in BBB permeability. Tumor margins were defined as the difference between the T_1_-enhancing area post-contrast agent injection and the area demarcated by a T_2_-weighted contrast outlining the tumor core [16]. The hypothesis was that an increased BBB permeability would facilitate the passage of the contrast agent, resulting in a more extensive diffusion beyond the tumor zone into the brain parenchyma. Consequently, an increase in the area of contrast uptake in the post-contrast T_1_-weighted image compared to the anatomical T_2_-weighted image would signify the opening of the BBB. Additionally, DCE-MRI analysis gave access to complementary tumor hemodynamic parameters that may be biomarkers of the change in BBB permeability [17]. These parameters were computed using two different models: the extended Tofts model [18] and the Patlak model [19]. The extended Tofts model is one of the most widely used in the literature for studying tumor perfusion/permeability in glioblastoma. It is a two-compartmental model suited to describe highly perfused tissues such as those found in glioblastoma. The Patlak model is comparable to the extended Tofts model but ignores the backflux and is therefore adapted to assess changes for a low-level BBB permeability where backflux is negligible, as found especially in the margins of glioblastoma tumors. This model may allow us to better assess, compared to the extended Tofts model, whether there are subtle changes in the hemodynamic parameters in tumor margins.

A diffusion-MRI study was also carried out. Diffusion-MRI emerges as a valuable tool in assessing the cytotoxic effects of medical treatments, particularly in the oncological domain. This imaging technique enables the study of water molecule movements within biological tissues without the use of an exogenous contrast agent, providing nuanced insights into cellular microstructure. One key parameter derived from diffusion–MRI is the Apparent Diffusion Coefficient (ADC). The ADC quantifies the ease with which water molecules move through tissues, offering valuable information about cellular density. In the context of cytotoxicity induced by treatment, the ADC can be used to define the microstructural changes associated with therapeutic response. Generally, an increase in ADC can be interpreted as an increase in water mobility, resulting in a decrease in cell density and indicating a cytotoxic response to the treatment [20,21,22].

For the histological study, we used Evans blue, a fluorescent marker of vascular leakage, to assess the functionality of the blood–brain barrier [23]. Indeed, administered intravenously, this dye forms a tight bond with plasma protein, albumin, in the bloodstream. Albumin, under normal circumstances, is too large to cross the intact BBB. However, in an event of disruption to the BBB, Evans blue bound to albumin could penetrate the brain parenchyma and be detected by fluorescence. Therefore, this approach enables us to determine a possible variation in tumor perfusion/permeability by analyzing the diffusion of Evans blue in the brain parenchyma.

## 2. Materials and Methods

The series of methods that have been used in the present study are similar to those used in a companion paper where we investigated the effect of a hyperosmolar shock on the integrity of the BBB by both histology and DCE-MRI. When the methods used are identical, the readers are invited to consult this companion paper [16].

### 2.1. Orthotopic U-87MG Mouse Model

All experiments were performed following the European Directive 2010/63/EU and following the Belgian national regulation guidelines. The protocols were approved by the ethical committee for animal care in the Health Sector of the UCLouvain (reference number of the file: 2022/UCL/MD/067). Water and food were given ad libitum. Animal body weight was monitored daily throughout the experiment. Six-week-old female NMRI nude mice (Janvier, France) were used. The tumor inoculation was carried out as previously described [16,24]. Tumor size monitoring was performed via MRI (see Section 2.3).

### 2.2. Irradiation Protocol

When the tumor size reached 7 ± 1 mm^3^ (as measured by MRI), mice were randomly assigned into 4 groups and irradiated once at doses of 0 (*n* = 5), 5 (*n* = 4), 10 (*n* = 4), and 15 Gy (*n* = 4) at a dose rate of 0.846 Gy/min with a Cesium-137 irradiator. A lead cover was placed over the mouse to protect the body from the irradiation. A hole in the lead cover allowed a 3 cm diameter irradiation field of the head of the mouse. The dosimetry was performed by Dr Kevin Souris (Molecular Imaging and Radiation Oncology research group, UCLouvain).

### 2.3. MRI

MRI was performed using a Bruker Biospec MRI system operating at 11.7 T (Bruker, Ettlingen, Germany). A quadrature transmit/receive birdcage coil (RAPID Biomedical, Rimpar, Germany) was used to image the brain of the mice which were anesthetized with isoflurane mixed with air (2.5% for induction and then 1.5% for maintenance). A heating blanket covered the mice. We monitored the temperature as well as the respiration rate of animals during the anesthesia.

Anatomical images were acquired using T_2_-weighted rapid acquisition using a protocol described previously [16]. Tumor volume was determined starting on day 14 following tumor induction, and then daily until the tumor size reached 7 ± 1 mm^3^ (the time at which tumors were irradiated). DCE-MRI images with a temporal resolution of 3 s were acquired using T_1_-weighted gradient echo images using acquisition parameters described previously [16]. DCE-MRI acquisitions were performed just before irradiation and 24 h and 48 h after irradiation. In addition, diffusion tensor imaging (DTI) was performed using an echoplanar imaging (EPI) sequence (echo time = 23.72 ms; repetition time = 4000 ms; segments = 4; field of view = 20 × 20 mm; matrix size = 128 × 128; resolution = 0.156 mm × 0.156 mm; slice thickness = 0.9 mm; acquisition time = 10 min 40 s) with diffusion sensitization in twelve directions and b values of 700, 1000, and 1500 s/mm^2^. Before DTI acquisition, an automatic shim correction was performed from the B0 map using the map_shim algorithm. Apparent Diffusion Coefficient (ADC) maps were computed. Diffusion MRI acquisitions were performed just before irradiation and 24 h and 48 h after irradiation.

### 2.4. Histological Analysis

The integrity of the BBB was assessed using the diffusion of the Evans blue (EB) dye in the brain parenchyma [25,26,27,28]. Evans blue was intravenously injected 48 h after the first irradiation (just after the last MRI experiment). The full detailed procedure has been described elsewhere [16].

### 2.5. Image Processing

Diffusion MRI data were analyzed using DSI studio (version 2017/01/27). The ADC parameter was measured in the tumor area. The tumor ROI was manually delineated on the T_2_-weighted anatomical images and was matched and positioned on the ADC maps.

We analyzed the DCE-MRI data using in-house software written in Matlab version R2021a. Different regions of interest (ROIs) were drawn as follows. ROI T_2_-weighted images allowed us to delineate the core of the tumor. The use of T_1_-weighted images after the injection of Gd-DOTA allowed us to delineate the area of the tumor in which the contrast agent was able to diffuse (called ROI T_1_). We also defined ROI Delta which corresponds to the difference between the ROI T_1_ from which we subtracted ROI T_2_. ROI Delta corresponds to the margins of the tumor. Using this procedure, we analyzed the evolution of hemodynamic parameters in the margins of the tumor and in the whole tumor.

The hemodynamic parameters were computed using two different pharmacokinetics models. The first one is the extended Tofts model [18] which is a two-compartmental model that describes a well-perfused tissue (glioblastoma are tumors that are regions that are enhanced after the injection of contrast agent). This model considers bidirectional transport between the blood plasma and the extracellular extravascular space (EES). The equation of this model is given by

(1) $$C_{t}\left(t\right)=v_{p}\cdot C_{p}\left(t\right)+K^{trans}\int_{0}^{t}C_{p}\left(\tau \right)e^{-k_{ep}(t-\tau )}d\tau$$

where *v_p_* is the blood plasma volume per unit volume of tissue, *K^trans^* is the transfer constant between blood plasma and EES [min^−1^], and *k_ep_* is the flux rate constant between EES and blood plasma [min^−1^] [29]. The EES volume per unit volume of tissue (*v_e_*) is calculated from the following equation:

(2) $$v_{e}=K^{trans}/k_{ep}$$

A second pharmacokinetic model was used: the Patlak model [19]. This model is comparable to the extended Tofts model but ignores the backflux from the EES into the blood plasma compartment and will therefore be applicable to see subtle changes for a low-level BBB permeability (as it is anticipated in tumor margins). Consequently, it only allows for the estimation of the two parameters *K^trans^* and *v_p_*. The equation of this model is given by

(3) $$C_{t}\left(t\right)=v_{p}\cdot C_{p}\left(t\right)+K^{trans}\int_{0}^{t}C_{p}\left(\tau \right)d\tau$$

We also evaluated AUC60 and AUC90 that correspond to areas under the curve (AUC) of contrast agent concentration as a function of time, from 0 to 60, or 0 to 90 s, respectively.

Histological sections were analyzed using the Qupath software (version 0.3.2) [28]. The histological sections (counterstained with diamidino-2-phenylindole (DAPI)) were examined under a fluorescence microscope slide scanner (Panoramic 250 Flash III, 3DHistech, Budapest, Hungary). DAPI and Cyanine 5 (Cy5) filters allowed us to delineate the tumor core and the region stained by the Evans blue dye.

### 2.6. Statistical Analyses

All data are expressed as means ± standard deviation (SD). All experiments were performed in triplicate or more. Two-way ANOVA tests (Tukey’s test) and *t*-tests were performed using GraphPad Prism (version 9.1.2), with *p*-values < 0.05 (*), *p* < 0.01 (**), *p* < 0.001 (***), and *p* < 0.0001 (****) considered as the levels of significance.

## 3. Results

### 3.1. The Diffusion of EB Dye Outside the Tumor Area Increased after 5 Gy, but Decreased at 10 and 15 Gy

**Figure 2:**
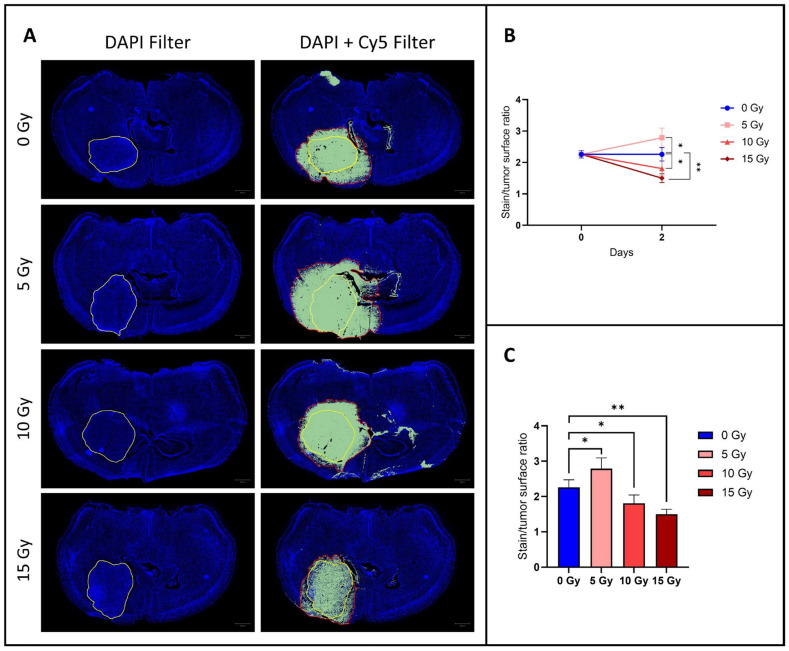
Evaluation of diffusion of contrast agent outside of the tumor area using histology with Evans blue dye. (**A**) Representative histological images of brain sections from a control mouse (*n =* 5) and irradiated mice with dosages of 5 Gy (*n* = 4), 10 Gy (*n* = 4), and 15 Gy (*n* = 4). The tumor area is encircled in yellow and the area of diffusion of EB is delineated in red. (**B**) Graph showing the ratio of the surface stained with EB on the tumor surface just before irradiation (day 0) and 24 h (day 1) and 48 h (day 2) after irradiation for the control group (blue) and group irradiated with 5 Gy (light red), 10 Gy (middle red), and 15 Gy (dark red). (**C**) Graph showing the same ratio as the top one for the same groups but focusing on day 2 after irradiation. Compared to the control group, the fluorescent dye diffused more widely outside the tumor area for an irradiation dose of 5 Gy, but diffused less widely for an irradiation dose of 10 Gy and even less widely for an irradiation dose of 15 Gy. The results are expressed as means ± SD. * *p*-values < 0.05, ** *p* < 0.01.

BBB integrity was assessed by perfusing mice with EB dye, a fluorescent vascular leakage marker. Compared to the control group, the fluorescent dye diffused more widely outside the tumor area for an irradiation dose of 5 Gy, but diffused less widely for an irradiation dose of 10 Gy and even less widely for an irradiation dose of 15 Gy (Figure 2). Indeed, there was a significant increase (*p* < 0.05) in the diffusion of the fluorescent marker outside the tumor (EB-stained/tumor surface ratio) for mice that received an irradiation dose of 5 Gy (2.79 ± 0.30, mean ± SD, *n* = 4) compared to the control group (2.26 ± 0.21, mean ± SD, *n* = 5). For a higher irradiation dose of 10 Gy, the EB-stained/tumor surface ratio (1.81 ± 0.24, mean ± SD, *n* = 4) was significantly lower (*p* < 0.05) compared to the control group, and for an even higher irradiation dose of 15 Gy, the EB-stained/tumor surface ratio (1.49 ± 0.14, mean ± SD, *n* = 4) was even more significantly lower (*p* < 0.01) than in the control group.

### 3.2. MRI Studies

#### 3.2.1. The ADC Value Increased after Irradiation: The Higher the Dose, the Higher the ADC

**Figure 3:**
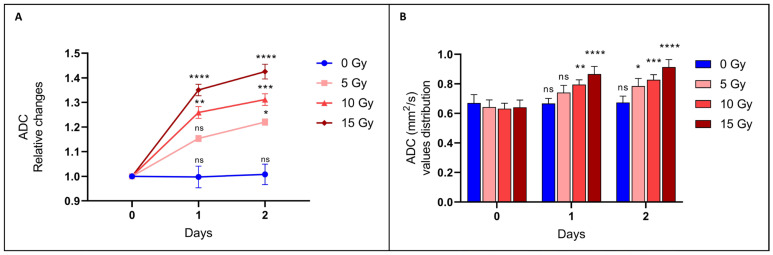
ADC measured by diffusion-MRI. Relative changes in ADC (**A**) and ADC values (**B**) between the control group (blue) and groups irradiated with 5 Gy (light red), 10 Gy (middle red), and 15 Gy (dark red). Diffusion-MRI acquisition was performed just before irradiation (day 0) and 24 h (day 1) and 48 h (day 2) after irradiation. On day 1, there was a significant increase in groups irradiated with 10 Gy (*n* = 4) and 15 Gy (*n* = 4) compared to the control group (*n* = 5). On day 2, there was a significant increase in the group irradiated with 5 Gy (*n* = 4) and an even more significant increase than on day 1 for groups irradiated with 10 Gy and 15 Gy. The higher the dose of irradiation used and the further in time after irradiation, the greater significant increase in ADC compared to the control group. The results are expressed as means ± SD. ns, non-significant, * *p*-values < 0.05, ** *p* < 0.01, *** *p* < 0.001, **** *p* < 0.0001.

The effect of irradiation on cell density, reflecting the cytotoxicity of the treatment, was assessed by monitoring the ADC obtained by diffusion-MRI. We observed an increase in ADC for irradiated mice and no change in ADC for control mice. The higher the dose of irradiation used and the later after irradiation, the greater the ADC increase compared to the control group (Figure 3). Indeed, there was a significant increase in the ADC on day 1 for mice irradiated at 10 Gy (0.80 ± 0.03, mean ± SD, *n* = 4, *p* < 0.01) and for mice irradiated at 15 Gy (0.87 ± 0.05, mean ± SD, *n* = 4, *p* < 0.0001) compared to the control group (0.66 ± 0.03, mean ± SD, *n* = 5), but there was no significant difference between mice irradiated at 5 Gy (0.74 ± 0.04, mean ± SD, *n* = 4) and the control group. On day 2, there was a significant increase for the group irradiated at 5 Gy (0.78 ± 0.05, mean ± SD, *p* < 0.05) compared to the control group (0.67 ± 0.04, mean ± SD) and an even more significant increase than on day 1 for the groups irradiated at 10 Gy (0.83 ± 0.03, mean ± SD, *p* < 0.001) and 15 Gy (0.91 ± 0.05, mean ± SD, *p* < 0.0001) compared to the control group. The increase in water mobility (higher ADC values) compared to the initial measurements (before treatment) suggest that there was a cytotoxic effect of irradiation on tumor tissue, with a decrease in cell density proportional to the increase in irradiation dose received.

#### 3.2.2. The T_1_/T_2_ Tumor Surface Ratio Did Not Change after Irradiation

**Figure 4:**
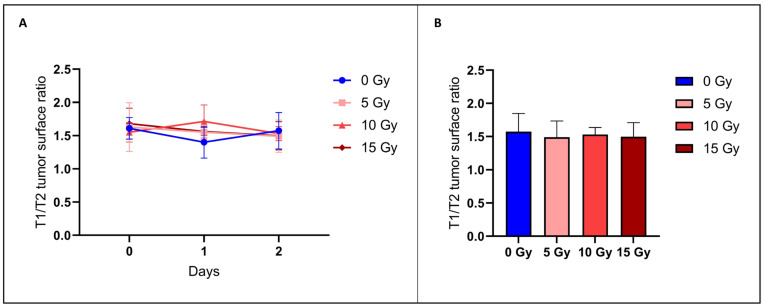
Assessment of diffusion of contrast agent outside of the tumor area using MRI. The left panel (**A**) shows the ratio between the T1 and T2 surface areas just before irradiation (day 0) and 24 h (day 1) and 48 h (day 2) after irradiation for the control group (blue) and group irradiated with 5 Gy (light red), 10 Gy (middle red), and 15 Gy (dark red). The right panel (**B**) shows the same ratio for the same groups focusing on day 2 after irradiation. Using this procedure, no significant difference in BBB permeability was observed between the control group (*n* = 5) and groups receiving 5 Gy (*n* = 4), 10 Gy (*n* = 4), or 15 Gy (*n* = 4). The results are expressed as means ± SD.

BBB permeability was additionally evaluated using MRI. Here, we determined the anatomical tumor size using T2-weighted contrast images and diffusion of the tracer inside and outside of the tumor using T1-weighted images after the administration of Gd-DOTA used as a contrast agent [16]. There was no significant difference (*p* > 0.05) between the T1/T2 surface ratio of the irradiated groups with 5 Gy (1.49 ± 0.24, mean ± SD, *n* = 4), 10 Gy (1.53 ± 0.10, mean ± SD, *n* = 4), or 15 Gy (1.49 ± 0.21, mean ± SD, *n* = 4) and the control group (1.57 ± 0.27, mean ± SD, *n* = 5) (Figure 4). These results indicate that this MRI assessment was unable to detect any difference in the BBB permeability between those groups.

#### 3.2.3. None of the Hemodynamic Parameters Measured by DCE-MRI Changed after Irradiation

**Figure 5:**
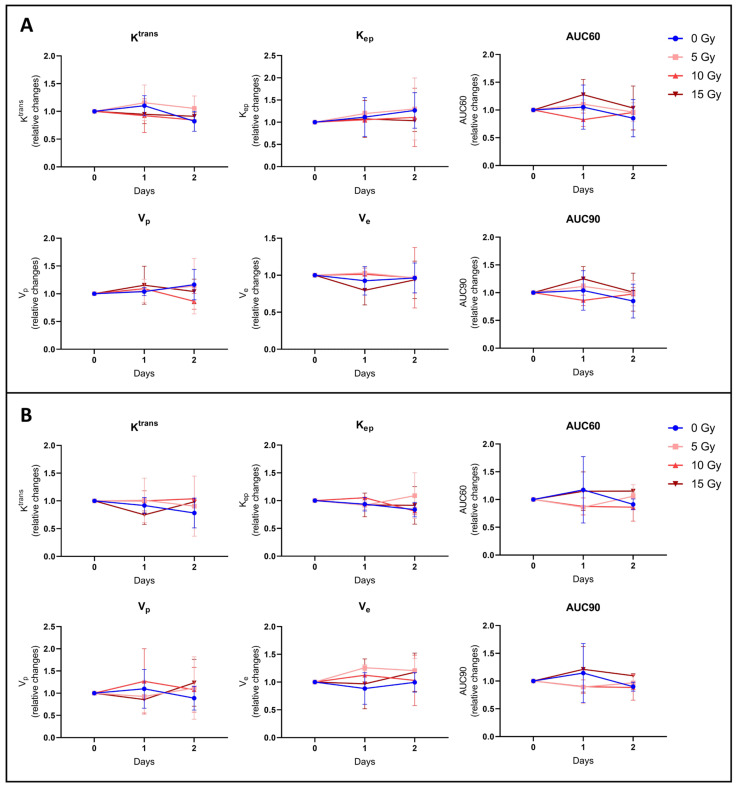
Hemodynamic parameters measured by DCE-MRI computed with extended Tofts pharmacokinetic model. Relative changes in tumor hemodynamic parameters between control group (blue), 5 Gy treated group (light red), 10 Gy treated group (middle red), and 15 Gy treated group (dark red) computed with Extended Tofts model using ROI T1 (**A**) corresponding to the whole tumor area and using ROI Delta (**B**) corresponding to the margin tumor area. There was no significant difference in the tumor hemodynamic parameters between the control group (*n* = 5) and groups receiving 5 Gy (*n* = 4), 10 Gy (*n* = 4), or 15 Gy (*n* = 4), whatever the studied parameter and ROI. The results are expressed as means ± SD.

**Figure 6:**
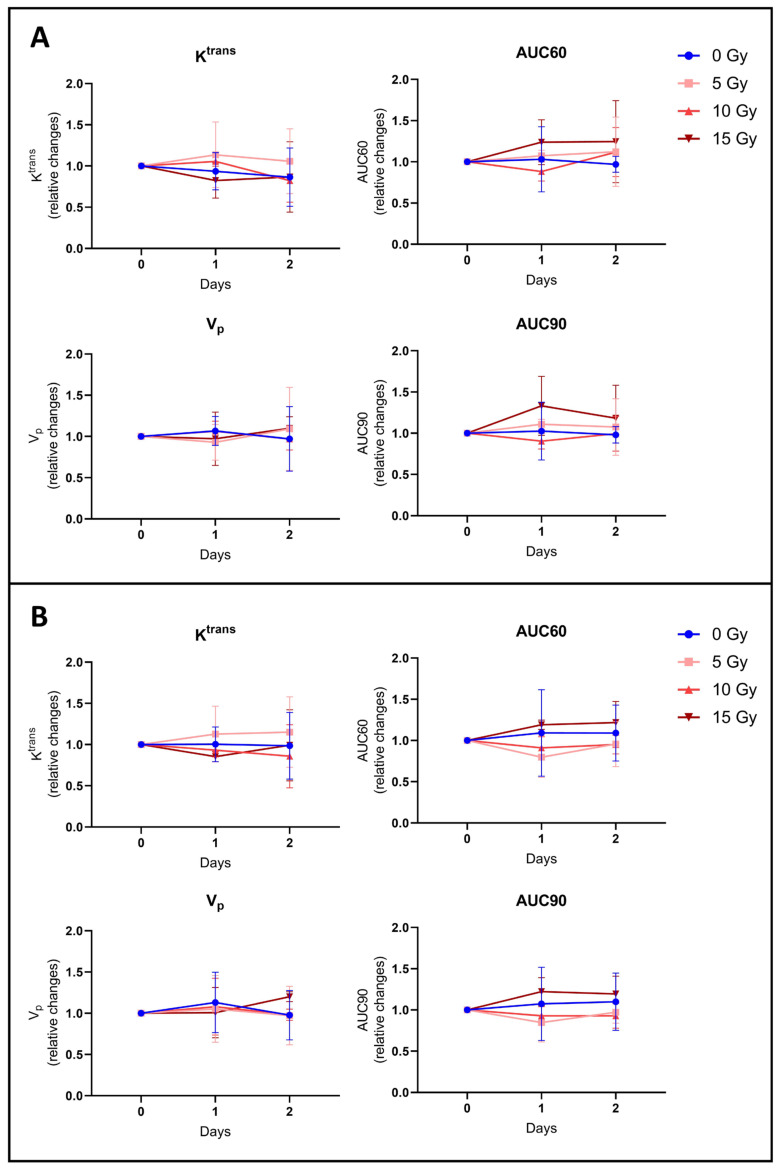
Hemodynamic parameters measured by DCE-MRI computed with Patlak pharmacokinetic model. Relative changes in tumor hemodynamic parameters between the control group (blue), 5 Gy treated group (light red), 10 Gy treated group (middle red), and 15 Gy treated group (dark red) computed with Patlak model using ROI T1 (**A**) corresponding to the whole tumor area and using ROI Delta (**B**) corresponding to the margin tumor area. DCE-MRI acquisition was performed just before irradiation (day 0) and 24 h (day 1) and 48 h (day 2) after irradiation. There was no significant difference in the tumor hemodynamic parameters between the control group (*n* = 5) and groups receiving 5 Gy (*n* = 4), 10 Gy (*n* = 4), or 15 Gy (*n* = 4), whatever the studied parameter and ROI. The results are expressed as means ± SD.

To further investigate BBB disruption, we also performed DCE-MRI to provide hemodynamic parameters such as the contrast agent efflux transfer constant (Ktrans), contrast agent reflux transfer constant (Kep), intravascular volume fraction (vp), extravascular volume fraction (ve), and area under the curve of contrast agent concentration as a function of time from 0 to 60 or 90 s (AUC60 or AUC90). These parameters were computed using two different models: the extended Tofts model and the Patlak model. These parameters were also analyzed in two different regions of interest (ROIs) of the tumor: ROI T1 corresponding to the whole tumor region, and ROI Delta corresponding to the margin tumor area. We did not observe any significant difference in the tumor hemodynamic parameters between the control group (*n* = 5) and groups receiving 5 Gy (*n* = 4), 10 Gy (*n* = 4), or 15 Gy (*n* = 4), whatever the studied parameter, model, and ROI used (Figure 5 and Figure 6).

**Figure 7:**
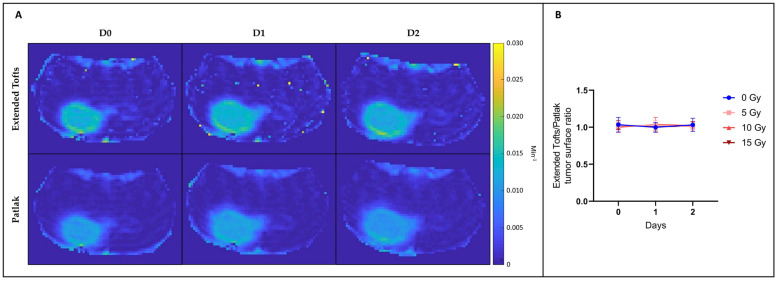
DCE-MRI parametric maps. (**A**) Illustration of typical K^trans^ maps obtained from 15 Gy irradiated mouse computed using extended Tofts model (top row) or Patlak model (bottom row). (**B**) The right panel shows the ratio between the tumor surface measured on the K^trans^ map from the extended Tofts model and the tumor surface measured on the K^trans^ map from the Patlak model. There is no significant difference between those tumor surface areas, whatever the irradiation dose used or the day after irradiation taken. The results are expressed as means ± SD.

We also compared the K^trans^ parametric maps between those computed using the extended Tofts model and those computed using the Patlak model (Figure 7). We calculated the ratio between the tumor surface measured on the K^trans^ map from the extended Tofts model and the tumor surface measured on the K^trans^ map from the Patlak model. We observed that this ratio was very close to 1, whatever the irradiation dose used or the day after irradiation. There was therefore no significant difference between tumor surface areas measured on K^trans^ maps obtained with the extended Tofts model and those obtained with the Patlak model.

## 4. Discussion

The heterogeneity of the BBB permeability in glioblastoma, with the BBB remaining intact in the infiltrative part of the tumor margins, limits the conventional systemic delivery of many chemotherapy drugs and allows residual tumor cells to escape cytotoxic treatments, leading to tumor recurrence [9,10,29,30]. These features underscore the need for innovative approaches to overcome this limited accessibility of drugs by achieving a homogeneous opening of the BBB [31]. In this article, we have evaluated if irradiation modulates the permeability of the BBB in glioblastoma, particularly in the margins of the tumor. To evaluate the impact of irradiation on the tumoral BBB, a multimodal approach involving MRI and histological analysis was employed. Evans blue is commonly used in preclinical studies as an indicator of vascular permeability, particularly to determine BBB disruption [32,33,34]. However, this histological method is highly invasive, requiring the sacrifice of the animal, and is therefore not suitable for clinical use. At first sight, contrast-enhanced MRI seems more attractive due to its non-invasive aspect and the fact that it is systematically used for the characterization of brain tumors in patients. Previous research has demonstrated the utility of contrast-enhanced MRI in preclinical models to examine changes in BBB permeability and assess the impact of strategies to open the BBB [35,36,37,38,39]. It should be pointed out that the majority of these studies have focused on the BBB opening in the brain parenchyma. While a series of studies have explored ultrasound-induced BBB opening in glioblastoma [40,41,42], none of them have, to our knowledge, examined the potential of DCE-MRI to monitor irradiation-induced BBB opening in glioblastoma. In this study, we focused on assessing the ability of irradiation to improve the delivery of compounds into the tumor margins. To achieve this objective, we compared the diffusion of BBB-impermeable agents into the brain parenchyma, for different irradiation doses.

The diffusion–MRI study allows us to assess the cytotoxic effects of irradiation by monitoring the Apparent Diffusion Coefficient (ADC). The results demonstrate a dose-dependent increase in the ADC, reflecting a decrease in cell density and indicating a cytotoxic response, especially at higher irradiation doses. This aligns with the expected response to irradiation [21].

Histological evaluation using Evans blue dye indicates a dose-dependent effect of irradiation on BBB integrity. Indeed, a significant increase in vascular leakage was observed for an irradiation dose of 5 Gy, followed by a significant reduction in leakage at higher irradiation doses (10 Gy and 15 Gy) (Figure 1). This suggests a complex relationship between irradiation dose and BBB disruption. We hypothesize that a low dose of irradiation will damage the tight junctions and endothelial cells without compromising the structural integrity of the vessel, resulting in an enhanced BBB permeability. Of course, we cannot exclude that irradiation could also affect other BBB components such as pericytes and astrocyte end feet. However, with a higher dose of irradiation, cytotoxicity could be so intense for the endothelial cells that the vessel would be destroyed, leading to a reduction in perfusion and diffusion. The decrease in the diffusion of the fluorescent dye in the brain parenchyma at high irradiation doses would therefore not be due to the BBB closing up but rather to the destruction of the vessel. Future work could clarify the contribution of these factors to the increase (at low irradiation dose) and decrease (at high irradiation dose) in permeability. Namely, histological studies with the labeling of CD31, alpha-SMA, desmin, and Glial Fibrillary Acidic Protein (GFAP) could highlight the evolution of endothelial cells, pericytes, and astrocytes. We can also add to this study that the area accessible to EB was 2.26 times larger than the anatomic area in the control group (Figure 1). This suggests that the BBB is already permeable in regions surrounding the bulk tumor in the U-87MG mouse glioblastoma model. 

In the MRI study, to determine the BBB permeability, we assessed the diffusion of a contrast agent outside the tumor area, as for the histological study, but here with gadolinium DOTA. To do so, we compared T_2_-weighted images (corresponding to the anatomical image of the bulk tumor) and T_1_-weighted images after Gd-DOTA administration (corresponding to areas accessible by the BBB-impermeable contrast agent). In the control group, the area accessible to Gd-DOTA visualized in the T_1_-weighted images was 57% larger than the bulk area of the tumor seen in the T_2_-weighted images (Figure 4). The area accessible to Gd-DOTA was not significantly altered for the irradiated mice whatever the dose of irradiation (Figure 4). Unlike the histological method, non-invasive MRI with Gd-DOTA appears unable to identify alterations in BBB permeability in adjacent tumor margins.

Regarding the DCE-MRI study, various models have been used to identify possible changes in hemodynamic parameters such as K^trans^, K_ep_, v_e_, v_p_, AUC60, and AU90 in the core and margins of the tumor. There were no significant changes in these parameters between control and irradiated groups whatever the parameter studied, the model used, or the area analyzed (Figure 5). Furthermore, we could have thought that with the Patlak model, which allows us to better assess a subtle change in BBB leakage of the poorly permeable tumor margin than with the extended Tofts model, we might have a slight difference between the tumor surface measured on the Patlak K^trans^ map and the tumor surface measured on the extended Tofts model K^trans^ map. However, as shown in Figure 6, there is no significant difference between those tumor surfaces.

In a previous work designed to evaluate the effect of an osmotic shock on the BBB permeability in a glioblastoma model, we observed the same mismatch between results from histology and DCE-MRI [16]. In this previous paper, we discussed that the disparate results observed between MRI and Evans blue staining to assess the opening of the tumoral BBB could be attributed to fundamental differences in the molecular and distribution properties of the contrast agents used for each technique. Indeed, contrary to Gd-DOTA, Evans blue (960 Da, molecular weight) is tightly bound to albumin. The consequence is that Evans blue bound to albumin can only cross the BBB when it is sufficiently compromised, whereas Gd-DOTA, because of its small size, can diffuse more easily into the brain parenchyma even through a weakly compromised BBB. As radiotherapy may damage the tight junctions between endothelial cells, we can assume that the observed change in permeability would be more pronounced for larger molecules (complex EB–albumin) than for smaller molecules (Gd-DOTA) that were already able to cross small fenestrations of the damaged tumoral BBB. Other reports have demonstrated differences in GBM uptake for small gadolinium complexes and ^124^I-labeled human serum albumin [43]. These variations in results highlight the importance of selecting the right contrast agent depending on the specific objective of the BBB study. For future studies, it would be interesting to test gadobenate (Gd-BOPTA) for this purpose, as this compound presents a high affinity for albumin, similar to Evans blue.

In addition, it is important to note that the values recorded via histology or MRI cannot be directly superimposed. Indeed, the invasive nature of the histological method requires the sacrifice of animals and, therefore, the images obtained in the control and treated groups came from different cohorts of animals, whereas the non-invasive MRI method enabled images measured longitudinally on the same animals to be obtained. It is also essential to point out that there are major differences in sensitivity and resolution between the two approaches. Indeed, the optical fluorescence method demonstrates superior sensitivity in the detection of probes, providing information on BBB permeability and offering significantly higher spatial resolution than the MRI technique.

The present study has some limitations. First, this study was carried out on a single glioblastoma model, the orthotopic U-87MG model grafted onto nude mice. This model has the advantage of high engraftment rates and good reproducibility. In addition, as the tumor core is well delineated, this model allows us to easily analyze the diffusion of compounds in the margins of the tumor. However, it is weakly infiltrative, which does not adequately represent the highly infiltrative biological properties of human glioblastoma [44]. Regarding the irradiation scheme, although three different incremental doses were administered to assess BBB permeability as a function of the increasing dose given, this administration was performed using a single dose. This is not representative of the fractionated or hypo-fractionated regimen that is used clinically for the treatment of newly diagnosed or recurrent glioblastoma [1,45]. Another limitation of this study is that we did not investigate the relevance of BBB irradiation opening when combined with different chemotherapies. The chemotherapy used in the standard-of-care TMZ would not have been relevant in this study, given that it already crosses the BBB very easily. Of note, a recent phase III trial showed that TMZ did not add any benefit in the treatment of IDH wildtype glioblastoma (new WHO 2021 classification) compared with radiotherapy alone, regardless of MGMT promoter status [46]. Considering this recent evidence and the fact that the benefit of the current approach is very limited, this should stimulate new research using other anti-cancer drugs for treating glioblastoma. For example, doxorubicin, paclitaxel, or albumin-paclitaxel (Abraxane) has shown interesting effects in pre-clinical models of glioblastoma [30,47,48], but the lack of BBB passage impedes reaching a sufficient therapeutic concentration at the tumor site [49,50]. In the future, it would be worth testing the effect of irradiation on the delivery of such chemotherapeutic agents.

## Data Availability

All data will be provided upon e-mail request.
